# Supplementary material for: Correlation between Antimicrobial Resistance and the Hospital-Wide Diverse Use of Broad-Spectrum Antibiotics by the Antimicrobial Stewardship Program in Japan
Source: Pharmaceutics. 2023 Feb 3;15(2):518. doi: 10.3390/pharmaceutics15020518 (PMC9964530; doi:10.3390/pharmaceutics15020518)
Supplement: Supplementary file 1 [file pharmaceutics-15-00518-s001.zip › pharmaceutics-2088965-supplementary.pdf]

Supplementary Materials:

Correlation between Antimicrobial Resistance and the Hospital-wide Diverse Use of Broad-Spectrum Antibiotics by the Antimicrobial Stewardship Program in Japan

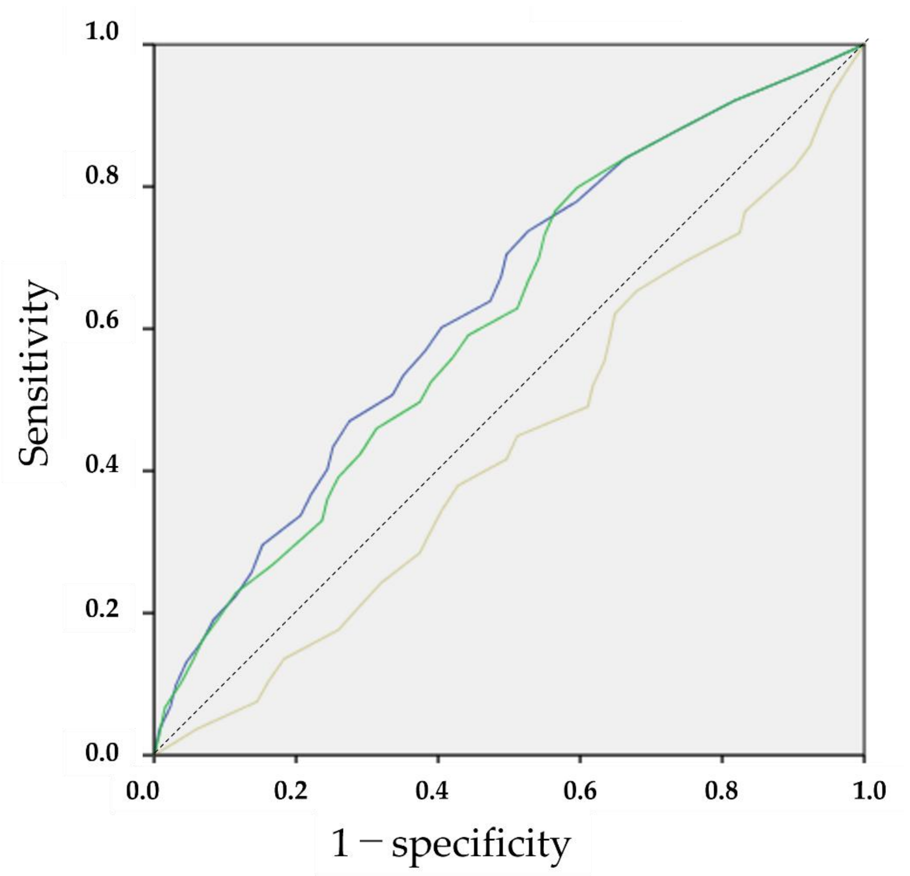

Figure S1. Receiver operating characteristic curve showing the detection ability for *Klebsiella pneumoniae* with resistance against ≥2 classes of antibiotics of the antibiotic heterogeneity index (AHI), the modified AHI, and the overall days of therapy/100 patient-days (DOT) for broad-spectrum antibiotics commonly used for hospital-onset infections. Blue line, AHI; green line, modified AHI; yellow line, DOT.

Table S1. Abbreviation list

AHI	Antibiotic heterogeneity index	ESBL	Extended-spectrum β-lactamase
AMR	Antimicrobial resistance	EUCAST	European Committee on Antimicrobial Susceptibility Testing
AS	Antibiotic stewardship	ICU	Intensive care unit
CLSI	Clinical and Laboratory Standards Institute	MIC	Minimum inhibitory concentration
CRE	Carbapenem-resistant Enterobacteriaceae	Modified AHI	Modified antibiotic heterogeneity index
CPE	Carbapenemase-producing Enterobacteriaceae	PAMS	Periodic monitoring and supervision
DOT	Days of therapy	SAAR	Standardized antimicrobial administration ratio

Table S2. Method of calculation for antibiotic heterogeneity indices

Antibiotic heterogeneity index (AHI)	AHI = 1 − {n/(2 × [n − 1])} × Σ ∣ ai − bi ∣<br>n: the number of antibiotic classes. n = 4 in this study.<br>ai: 0.25 for each antibiotic class when 4 antibiotic classes are equally used<br>bi: the proportion of observed day of therapy (DOT) for each antibiotic class<br><br>1. When the observed proportion of DOT (% DOT) is 25% in carbapenems, 25% in piperacillin/tazobactam, 25% in cephalosporins/ceftazidime/aztreonam and 25% in fluoroquinolones, "AHI = 1" is achieved.<br>2. When the proportion of DOT is 40% in carbapenems, 30% in piperacillin/tazobactam, 20% in cephalosporins/ceftazidime/aztreonam and 10% in fluoroquinolones, AHI is calculated as follows;<br>**AHI = 1 − {4/(2 × [4 − 1])} × (∣ 0.4 − 0.25 ∣ + ∣ 0.3 − 0.25 ∣ + ∣ 0.2 − 0.25 ∣ + ∣ 0.1 − 0.25 ∣)= 0.733**
Modified AHI	n = 4<br>ai: 0.1 for fluoroquinolones and 0.3 for remaining 3 antibiotic classes<br><br>1. When the observed %DOT is 30% in carbapenems, 30% in piperacillin/tazobactam, 30% in cephalosporins/ceftazidime/aztreonam and 10% in fluoroquinolones, "modified AHI = 1" is achieved.<br>2. When the observed %DOT is 40% in carbapenems, 30% in piperacillin/tazobactam, 20% in cephalosporins/ceftazidime/aztreonam and 10% in fluoroquinolones, AHI is calculated as follows;<br>**AHI = 1 − {4/(2 × [4 − 1])} × (∣ 0.4 − 0.3 ∣ + ∣ 0.3 − 0.3 ∣ + ∣ 0.2 − 0.3 ∣ + ∣ 0.1 − 0.1 ∣) = 0.866**

Table S3. MIC breakpoints based on EUCAST and the CLSI in *Pseudomonas aeruginosa*, *Acinetobacter baumannii*, and Enterobacteriaceae spp.

Organisms	Antimicrobial agent	MIC breakpoint (μg/mL)	
		CLSI 2018 Resistant/intermediate susceptibility	EUCAST 2023 Resistance
Pseudomonas aeruginosa	Meropenem	≥4	≥16
	Piperacillin/tazobactam	≥32	≥32
	Cefepime	≥16	≥16
	Ciprofloxacin	≥2	≥1
	Amikacin	≥32	≥32
	Gentamicin	≥8	–
Acinetobacter baumannii	Meropenem	≥4	≥16
	Piperacillin/tazobactam	≥32	–*
	Cefepime	≥16	–*
	Ciprofloxacin	≥2	≥2
	Amikacin	≥32	≥16
	Gentamicin	≥8	≥8
Enterobacteriaceae spp.	Meropenem	≥2	≥16
	Piperacillin/tazobactam	≥32	≥16
	Cefepime	≥4	≥8
	Levofloxacin	≥4	≥2
	Amikacin	≥32	≥16
	Gentamicin	≥8	≥4

MIC: minimum inhibitory concentration; EUCAST: European committee on antimicrobial susceptibility testing; CLSI: the Clinical and Laboratory Standards Institute; *: Criteria for *Pseudomonas aeruginosa* were used

Table S4. Patient background information for each year between April 2015 and March 2022.

**Factors**	**2015 Apr.–2016 Mar.**	**2016 Apr.–2017 Mar.**	**2017 Apr.–2018 Mar.**	**2018 Apr.–2019 Mar.**	**2019 Apr.–2020 Mar.**	**2020 Apr.–2021 Mar.**	**2021 Apr.–2022 Mar.**
Hospitalization	22,820	23,303	23,866	23,863	24,415	21,931	21,264
Number of patients undergoing surgery (rate)	6519 (28.6%)	6465 (27.7%)	6567 (27.5%)	6431 (26.9%)	6510 (26.7%)	6117 (27.9%)	6090 (28.6%)
Male（%）	11849 (51.9%)	12231 (52.5%)	12406 (52.0%)	12359 (51.8%)	12557 (51.4%)	11351 (51.8%)	10893 (51.2%)
Age							
0−9 years	8.4%	8.4%	8.3%	8.2%	8.0%	8.1%	8.6%
10−19 years	4.0%	3.7%	3.5%	3.4%	3.7%	3.7%	3.5%
20−29 years	4.7%	4.6%	4.3%	4.2%	4.0%	4.1%	4.4%
30−39 years	7.8%	7.4%	7.0%	6.8%	6.8%	6.7%	7.0%
40−49 years	9.6%	9.8%	9.6%	9.8%	9.3%	9.4%	9.0%
50−59 years	11.0%	10.8%	11.2%	11.4%	11.3%	11.9%	12.1%
60−69 years	19.7%	19.9%	18.8%	17.4%	16.2%	15.7%	15.0%
70−79 years	22.7%	22.5%	23.5%	24.5%	25.4%	25.1%	24.6%
80−89 years	10.8%	11.7%	12.6%	12.8%	13.6%	13.4%	14.2%
>90 years	1.1%	1.2%	1.1%	1.5%	1.6%	1.8%	1.6%
Patient-days	280,378	284,665	283,285	278,550	283,669	265,000	254,776
Cardiovascular medicine	13,129 (4.7%)	14,410 (5.1%)	14,756 (5.2%)	16,544 (5.9%)	16,040 (5.7%)	15,992 (6.0%)	15,043 (5.9%)
Hematology	14,532 (5.2%)	16,624 (5.8%)	16,784 (5.9%)	16,390 (5.9%)	15,434 (5.4%)	15,632 (5.9%)	14,797 (5.8%)
Allergy and rheumatology	5,777 (2.1%)	6,294 (2.2%)	7,205 (2.5%)	6,300 (2.3%)	5,090 (1.8%)	4,995 (1.9%)	4,228 (1.7%)
Diabetes, metabolism, and endocrinology	8,643 (3.1%)	9,000 (3.2%)	9,484 (3.3%)	8,030 (2.9%)	7,502 (2.6%)	6,978 (2.6%)	6,357 (2.5%)
Gastroenterology	31.470 (11.2%)	32,205 (11.3%)	31,833 (11.2%)	31,608 (11.3%)	31,300 (11.0%)	28,486 (10.7%)	26,667 (10.5%)
Respiratory medicine	16,506 (5.9%)	15,712 (5.5%)	16,097 (5.7%)	16,341 (5.9%)	16,179 (5.7%)	15,338 (5.8%)	15,226 (6.0%)
Neurology	6,771 (2.4%)	7,120 (2.5%)	7,420 (2.6%)	6,751 (2.4%)	7,650 (2.7%)	6,258 (2.4%)	6,070 (2.4%)
Nephrology and artificial dialysis	7,155 (2.6%)	7,477 (2.6%)	7,637 (2.7%)	7,118 (2.6%)	7,643 (2.7%)	7,591 (2.9%)	7,032 (2.8%)
General medicine	0 (0.0%)	0 (0.0%)	0 (0.0%)	631 (0.2%)	896 (0.3%)	0 (0.0%)	0 (0.0%)
Pediatrics	5,591 (2.0%)	5,413 (1.9%)	5,363 (1.9%)	4,166 (1.5%)	4,794 (1.7%)	3,637 (1.4%)	4,618 (1.8%)
Neonatal intensive care unit	10,175 (3.6%)	8,395 (2.9%)	6,549 (2.3%)	7,555 (2.7%)	6,356 (2.2%)	6,919 (2.6%)	6,928 (2.7%)
Pediatric surgery	954 (0.3%)	1,102 (0.4%)	1,168 (0.4%)	964 (0.3%)	872 (0.3%)	1,361 (0.5%)	937 (0.4%)
Gastroenterological surgery	31,380 (11.2%)	30,553 (10.7%)	28,656 (10.1%)	27,427 (9.8%)	29,102 (10.3%)	27,314 (10.3%)	27,279 (10.7%)
Breast and endocrine surgery	4,593 (1.6%)	4,398 (1.5%)	3,829 (1.4%)	3,677 (1.3%)	3,302 (1.2%)	3,324 (1.3%)	2,998 (1.2%)
Cardiovascular surgery	6,359 (2.3%)	6,129 (2.2%)	6,237 (2.2%)	6,984 (2.5%)	7,887 (2.8%)	7,070 (2.7%)	5,783 (2.3%)
Thoracic surgery	7,751 (2.8%)	6,886 (2.4%)	7,193 (2.5%)	7,250 (2.6%)	8,598 (3.0%)	7,870 (3.0%)	7,961 (3.1%)
Orthopedic surgery	20,022 (7.1%)	20,088 (7.1%)	19,563 (6.9%)	19,369 (7.0%)	19,606 (6.9%)	17,224 (6.5%)	16,361 (6.4%)
Plastic and reconstructive surgery	2,799 (1.0%)	2,189 (0.8%)	1,709 (0.6%)	2,039 (0.7%)	1,997 (0.7%)	1,449 (0.5%)	1,860 (0.7%)
Neurosurgery	16,081 (5.7%)	17,944 (6.3%)	21,987 (7.8%)	17,912 (6.4%)	21,375 (7.5%)	20,662 (7.8%)	21,317 (8.4%)
Dermatology	1,813 (0.6%)	2,379 (0.8%)	2,829 (1.0%)	2,483 (0.9%)	2,466 (0.9%)	2,058 (0.8%)	2,097 (0.8%)
Urology	10,116 (3.6%)	9,505 (3.3%)	9,918 (3.5%)	10,881 (3.9%)	10,193 (3.6%)	10,385 (3.9%)	9,557 (3.8%)
Obstetrics and gynecology	11,767 (4.2%)	10,873 (3.8%)	11,059 (3.9%)	10,403 (3.7%)	11,832 (4.2%)	11,383 (4.3%)	9,980 (3.9%)
Ophthalmology	11,832 (4.2%)	12,065 (4.2%)	11,342 (4.0%)	10,944 (3.9%)	10,758 (3.8%)	10,038 (3.8%)	9,908 (3.9%)
Otorhinolaryngology	9,532 (3.4%)	10,510 (3.7%)	8,711 (3.1%)	9,534 (3.4%)	9,389 (3.3%)	7,924 (3.0%)	9,645 (3.8%)
Radiology	1,396 (0.5%)	1,832 (0.6%)	1,605 (0.6%)	1,341 (0.5%)	1,229 (0.4%)	1,146 (0.4%)	973 (0.4%)
Dentistry and oral surgery	3,601 (1.3%)	3,281 (1.2%)	2,807 (1.0%)	3,918 (1.4%)	3,398 (1.2%)	3,094 (1.2%)	3,222 (1.3%)
Anesthesiology and pain relief center	856 (0.3%)	892 (0.3%)	708 (0.2%)	1,156 (0.4%)	1,053 (0.4%)	1,090 (0.4%)	1,453 (0.6%)
Emergency and critical care medicine	8,780 (3.1%)	8,468 (3.0%)	9,197 (3.2%)	9,904 (3.6%)	11,385 (4.0%)	9,164 (3.5%)	9,137 (3.6%)
Neuropsychiatry	10,997 (3.9%)	12,921 (4.5%)	11,639 (4.1%)	10,930 (3.9%)	10,343 (3.6%)	10,618 (4.0%)	7,342 (2.9%)
Intensive care unit*	4,068 (1.5%)	4,036 (1.4%)	4,272 (1.5%)	3,990 (1.4%)	4,160 (1.5%)	3,842 (1.4%)	3,793 (1.5%)

*: Patients in intensive care unit belongs to each department in the university hospital.

Table S5. Standardized antimicrobial administration ratio of adult broad-spectrum antibacterial agents predominantly used for hospital-onset infections in the Department of Respiratory Medicine, the Department of Colorectal Surgery, and the intensive care unit

**Department**	**Standardized antimicrobial administration ratio**						
	April 2015 to March 2016	April 2016 to March 2017	April 2017 to March 2018	April 2018 to March 2019	April 2019 to March 2020	April 2020 to March 2021	April 2021 to March 2022
Intensive care unit	1.633	1.819	1.693	1.440	1.251	1.100	1.283
Respiratory medicine	0.504	0.747	0.885	0.880	1.037	1.008	1.008
Colorectal surgery	0.985	1.204	1.105	0.912	0.929	0.699	1.065

Table S6. Annual change in the isolation rates of carbapenemase-producing Enterobacteriaceae and carbapenem-resistant isolates in Enterobacte-riaceae sp., *Pseudomonas aeruginosa*, and *Acinetobacter baumannii*

**Organisms**	**Carbapenem Resistant organisms**	**2015 Apr.– 2016 Mar.**	**2016 Apr.– 2017 Mar.**	**2017 Apr.– 2018 Mar.**	**2018 Apr.– 2019 Mar.**	**2019 Apr.– 2020 Mar.**	**2020 Apr.– 2021 Mar.**	**2021 Apr.– 2022 Mar.**	**Total**
Enterobacteriaceae spp.	Carbapenemase-producing Enterobacteriaceae	12/1099 (1.1%)	4/1022 (0.4%)	0/902 (0.0%)	1/988 (0.1%)	2/1179 (0.2%)	3/1313 (0.2%)	1/1335 (0.1%)	23/7838 (0.3%)
	Carbapenem-resistant Enterobacteriaceae	1/1099 (0.1%)	2/1022 (0.2%)	1/902 (0.1%)	5/988 (0.5%)	3/1179 (0.3%)	1/1313 (0.1%)	1/1335 (0.1%)	14/7838 (0.2%)
Pseudomonas aeruginosa	Carbapenem-resistant *Pseudomonas aeruginosa*	15/184 (8.2%)	7/183 (3.8%)	7/170 (4.1%)	13/172 (7.6%)	16/211 (7.6%)	15/205 (7.3%)	32/267 (12.0%)	105/1392 (7.5%)
Acinetobacter baumannii	Carbapenem-resistant *Acinetobacter baumannii*	0/51 (0.0%)	0/68 (0.0%)	1/44 (2.3%)	0/25 (0.0%)	0/47 (0.0%)	1/48 (2.1%)	1/38 (2.6%)	3/321 (0.9%)

Table S7. Resistance rates against antibiotics with anti-*Pseudomonas* activity in glucose non-fermenting Gram-negative rods and Enterobacteriaceae spp.

**Organisms**	**No. of strains**	**No. of resistant organisms against antibiotics with anti-pseudomonas activity**				
		Meropenem (%)	Piperacillin/tazobactam (%)	Cefepime (%)	Ciprofloxacin for glucose non-fermenting Gram-negative rods; levofloxacin for Enterobacteriaceae (%)	Gentamicin or amikacin (%)
Glucose non-fermenting Gram-negative rods	1713	108 (6.3%)	231 (13.5%)	240 (14.0%)	274 (16.0%)	55 (3.2%)
Pseudomonas aeruginosa	1392	105 (7.5%)	183 (13.1%)	224 (16.1%)	253 (18.2%)	36 (2.6%)*
Acinetobacter baumannii	321	3 (0.9%)	48 (15.0%)	16 (5.0%)	21 (6.5%)	19 (5.9%)
Enterobacteriaceae spp.	7838	14 (0.2%)	526 (6.7%)	1000 (12.8%)	1441 (18.4%)	630 (8.0%)
Escherichia coli	2863	0 (0.0%)	90 (3.1%)	671 (23.4%)	1121 (39.2%)	412 (14.4%)
Klebsiella pneumoniae	1685	7 (0.4%)	82 (4.9%)	158 (9.4%)	124 (7.4%)	114 (6.8%)
Klebsiella oxytoca	670	1 (0.1%)	47 (7.0%)	21 (3.1%)	23 (3.4%)	9 (1.3%)
Klebsiella aerogenes	339	2 (0.6%)	54 (15.9%)	15 (4.4%)	11 (3.2%)	5 (1.5%)
Enterobacter cloacae	960	2 (0.2%)	213 (22.2%)	101 (10.5%)	71 (7.4%)	21 (2.2%)
Other Enterobacteriaceae	1321	2 (0.2%)	40 (3.0%)	34 (2.6%)	91 (6.9%)	69 (5.2%)
Serratia marcescens	382	0 (0.0%)	17 (4.5%)	5 (1.3%)	13 (3.4%)	101 (26.4%)
Citrobacter freundii	300	2 (0.7%)	11 (3.7%)	10 (3.3%)	33 (11.0%)	10 (3.3%)
Proteus mirabilis	204	0 (0.0%)	0 (0.0%)	11 (5.4%)	37 (18.1%)	23 (11.3%)
Proteus vulgaris	140	0 (0.0%)	3 (2.1%)	2 (1.4%)	3 (2.1%)	5 (3.6%)
Morganella morganii	295	0 (0.0%)	9 (3.1%)	6 (2.0%)	5 (1.7%)	21 (7.1%)

*: Resistance only for amikacin was evaluated in the resistance rate against aminoglycosides in *Pseudomonas aeruginosa*

Table S8. Isolation of resistant or intermediate susceptibility organisms based on CLSI criteria in glucose non-fermenting Gram-negative rods and Enterobacteriaceae spp.

**Organisms**	**No. of strains**	**No. of resistant or intermediate susceptibility organisms (rate)**		**No. of resistant organisms against antibiotics with anti-pseudomonas activity**				
		≥2 classes	≥3 classes	Meropenem (%)	Piperacillin/tazobactam (%)	Cefepime (%)	Ciprofloxacin for glucose non-fermenting Gram-negative rods; levofloxacin for Enterobacteriaceae (%)	Gentamicin or amikacin (%)
Glucose non-fermenting Gram-negative rods	1713	293 (17.1%)	165 (9.6%)	241 (14.1%)	231 (13.5%)	240 (14.0%)	230 (13.4%)	264 (15.4%)
Pseudomonas aeruginosa	1392	270 (19.4%)	159 (11.4%)	233 (16.7%)	183 (13.1%)	224 (16.1%)	209 (15.0%)	245 (17.6%)
Acinetobacter baumannii	321	23 (7.2%)	6 (1.9%)	8 (2.5%)	48 (15.0%)	16 (5.0%)	21 (6.5%)	19 (5.9%)
Enterobacteriaceae spp.	7838	1082 (13.8%)	284 (3.6%)	45 (0.6%)	478 (6.1%)	1053 (13.4%)	1343 (17.1%)	495 (6.3%)
Escherichia coli	2863	771 (26.9%)	173 (6.0%)	6 (0.2%)	86 (3.0%)	708 (24.7%)	1109 (38.7%)	329 (11.5%)
Klebsiella pneumonia	1685	131 (7.8%)	80 (4.7%)	19 (1.1%)	65 (3.9%)	165 (9.8%)	113 (6.7%)	104 (6.2%)
Klebsiella oxytoca	670	25 (3.7%)	10 (1.5%)	1 (0.1%)	46 (6.9%)	24 (3.6%)	19 (2.8%)	9 (1.3%)
Klebsiella aerogenes	339	14 (4.1%)	6 (1.8%)	5 (1.5%)	49 (14.5%)	15 (4.4%)	5 (1.5%)	4 (1.2%)
Enterobacter cloacae	960	111 (11.6%)	9 (0.9%)	8 (0.8%)	195 (20.3%)	105 (10.9%)	45 (4.7%)	9 (0.9%)
Other Enterobacteriaceae spp.	1321	30 (2.3%)	6 (0.5%)	6 (0.5%)	37 (2.8%)	36 (2.7%)	52 (3.9%)	40 (3.0%)
Serratia marcescens	382	3 (0.8%)	1 (0.3%)	2 (0.5%)	15 (3.9%)	5 (1.3%)	5 (1.3%)	6 (1.6%)
Citrobacter freundii	300	11 (3.7%)	4 (1.3%)	4 (1.3%)	11 (3.7%)	10 (3.3%)	19 (6.3%)	8 (2.7%)
Proteus mirabilis	204	5 (2.5%)	0 (0.0%)	0 (0.0%)	0 (0.0%)	12 (5.9%)	24 (11.8%)	8 (3.9%)
Proteus vulgaris	140	3 (2.1%)	0 (0.0%)	0 (0.0%)	2 (1.4%)	2 (1.4%)	1 (0.7%)	1 (0.7%)
Morganella morganii	295	8 (2.7%)	1 (0.3%)	0 (0.0%)	9 (3.1%)	7 (2.4%)	3 (1.0%)	17 (5.8%)

Table S9. Correlation between the quarterly antimicrobial heterogeneity index (AHI), modified AHI, and days of therapy per 100 patient-days for broad-spectrum antibiotics, and the quarterly rate of resistant organisms among glucose non-fermenting Gram-negative rods and Enterobacteriaceae spp. between April 2015 and March 2022

	**Antimicrobial heterogeneity index**				**Modified antimicrobial heterogeneity index**				**DOT (days of therapy per 100 patient-days)**			
	**Gradient**	**R^2**	***p*-Value**	**95% CI**	**Gradient**	**R^2**	***p*-Value**	**95% CI**	**Gradient**	**R^2**	***p*-Value**	**95% CI**
Glucose non-fermenting Gram-negative rods												
≥ 2 antibiotic classes	−0.391	0.176	0.026	−0.685 to −0.054	−0.557	0.320	0.002	−0.775 to −0.245	0.003	0.005	0.727	−0.312 to 0.431
≥ 3 antibiotic classes	−0.337	0.166	0.031	−0.678 to −0.041	−0.478	0.302	0.002	−0.766 to −0.222	0.001	0.000	0.950	−0.362 to 0.384
Enterobacteriaceae spp.												
≥ 2 antibiotic classes	−0.253	0.208	0.015	−0.708 to −0.099	−0.310	0.280	0.004	−0.753 to −0.194	−0.001	0.001	0.908	−0.393 to 0.353
≥ 3 antibiotic classes	−0.045	0.020	0.476	−0.488 to 0.245	−0.060	0.031	0.372	−0.515 to 0.211	0.000	0.000	0.948	−0.384 to 0.362

95% CI: 95% confidence interval
